# Classification of Metastatic and Non-Metastatic Thoracic Lymph Nodes in Lung Cancer Patients Based on Dielectric Properties Using Adaptive Probabilistic Neural Networks

**DOI:** 10.3389/fonc.2021.640804

**Published:** 2021-03-05

**Authors:** Di Lu, Hongfeng Yu, Zhizhi Wang, Zhiming Chen, Jiayang Fan, Xiguang Liu, Jianxue Zhai, Hua Wu, Xuefei Yu, Kaican Cai

**Affiliations:** ^1^ Department of Thoracic Surgery, Nanfang Hospital, Southern Medical University, Guangzhou, China; ^2^ School of Biomedical Engineering, Southern Medical University, Guangzhou, China

**Keywords:** dielectric properties, thoracic lymph nodes, simulated annealing algorithm, probabilistic neural network, metastatic

## Abstract

**Objective:**

Dielectric properties can be used in normal and malignant tissue identification, which requires an effective classifier because of the high throughput nature of the data. With easy training and fast convergence, probabilistic neural networks (PNNs) are widely applied in pattern classification problems. This study aims to propose a classifier to identify metastatic and non-metastatic thoracic lymph nodes in lung cancer patients based on dielectric properties.

**Methods:**

The dielectric properties (permittivity and conductivity) of lymph nodes were measured using an open-ended coaxial probe. The Synthetic Minority Oversampling Technique method was adopted to modify the dataset. Feature parameters were scored to select the appropriate feature vector using a Statistical Dependency algorithm. The dataset was classified using adaptive PNNs with an optimized smooth factor using the simulated annealing PNN (SA-PNN). The results were compared with traditional Probabilistic, Support Vector Machines, k-Nearest Neighbor and the Classify functions in MATLAB.

**Results:**

The conductivity frequencies of 3959, 3958, 3960, 3978, 3510, 3889, 3888, and 3976 MHz were selected as the feature vectors for 219 lymph nodes (178 non-metastatic and 41 metastatic). Compared with the other methods, SA-PNN achieved the highest classification accuracy (92.92%) and the corresponding specificity and sensitivity were 94.72% and 91.11%, respectively.

**Conclusions:**

Compared with the other methods, the SA-PNN proposed in the present study achieved a higher classification accuracy, which provides a new scheme for classification of metastatic and non-metastatic thoracic lymph nodes in lung cancer patients based on dielectric properties.

## Introduction

Dielectric properties usually include effective dielectric permittivity and conductivity (1), which are intrinsic properties of biological tissues and can indirectly reflect the physiological state changes of tissues. Previous studies have reported that dielectric properties could be used as an index parameter for identification of normal and malignant liver (2), thyroid (3), breast (4), and colorectal tissues (5). For the measured dielectric data, the dielectric parameters at each frequency point are equivalent to the feature parameters. This makes the data cumbersome and reduces the classification efficiency. Therefore, it is necessary to select an effective classifier for abnormal tissue identification.

Probabilistic neural network (PNN) was first proposed by Donald F. Specht in the late 18^th^ century. The theoretical basis of the network is Bayesian classification theory and probability density function estimation (6–10). It can realize the function of nonlinear learning algorithms with linear learning algorithms, which is widely applied in pattern classification problems. Compared with other neural networks, PNN has the advantages of easy training and fast convergence. Therefore, it is suitable for real-time classification. In PNN, the radial basis function in the pattern layer transfers data as an activation function, and the smooth factor σ determines the width of the Gaussian curve (9). However, in the traditional PNN, the value of σ of each neuron in the pattern layer is fixed, which leads to the failure to fully reflect the real situation of the sample space and limits the performance of the network. Therefore, allowing activation functions of different neuron classes in the pattern layer to take different σ values will improve the performance of the network. Simulated annealing (SA) algorithm is a general optimization algorithm based on probability. It can find the optimal solution of the objective function in a large space. It has the advantages of strong robustness, is suitable for parallel processing, and can be used for the optimization of complex nonlinear problems (11, 12).

In this study, an adaptive probabilistic neural network with an optimized smooth factor by simulated annealing algorithm (SA-PNN) is proposed to classify metastatic and non-metastatic thoracic lymph nodes in lung cancer patients based on dielectric properties. Expected classification results are obtained.

## Materials and Methods

### Data Introduction

The dielectric parameters of lymph nodes were measured using open-ended coaxial probes (13, 14). All measurements were from patients receiving lung surgery in the Department of Thoracic Surgery, Nanfang Hospital, Southern Medical University. All thoracic lymph nodes were freshly obtained during surgery within 10 min after these samples were removed from the patients to increase the time-sensitivity to increase the time-sensitivity. The metastatic status of the thoracic lymph nodes was determined by regular pathological examination. Related human tissue studies were approved by the ethics committee of Nanfang Hospital, Southern Medical University, Guangzhou, China (NFEC-2017-070). This trial was registered at https://clinicaltrials.gov (registration number: NCT03339479) and all patients provided informed consent in accordance with the Declaration of Helsinki. The Synthetic Minority Oversampling Technique (SMOTE) algorithm was used to preprocess the lymph node data (15).

### Feature Parameter Scoring

The Statistical Dependency (SD) method is applied in this paper as the feature scoring algorithm to score the permittivity and conductivity at each frequency point. As a feature scoring algorithm, the goal of the SD method is simply to measure whether the values of a feature are dependent on the associated class labels or whether the two simply co-occur by chance. The statistical dependence between the discretized feature values y and the class labels z is evaluated according to Formula (1) (16):

(1) $$SD=\sum_{y\in Y}\sum_{z\in Z}p\left(y,z\right)\frac{p\left(y,z\right)}{p\left(y\right)p\left(z\right)}$$

In this formula, p(y, z) stands for the joint probability distribution of y and z, p(y) and p(z) stand for the marginal probability distribution functions of y and z, respectively. The larger the SD, the higher the dependency between the feature values and the class labels. In the case that the feature is fully independent of the class labels, the SD will obtain the minimal value of one. The SD value of each feature parameter can be obtained by calculating them using this formula. The SD value minus one (SD-1) was taken as the final score of the feature parameters.

Different feature subsets are combined for classification experiments. The feature subset with the best classification result is selected as the final feature vector for identification and classification.

### Probabilistic Neural Network

#### Probabilistic Neural Network Model

**Figure 1:**
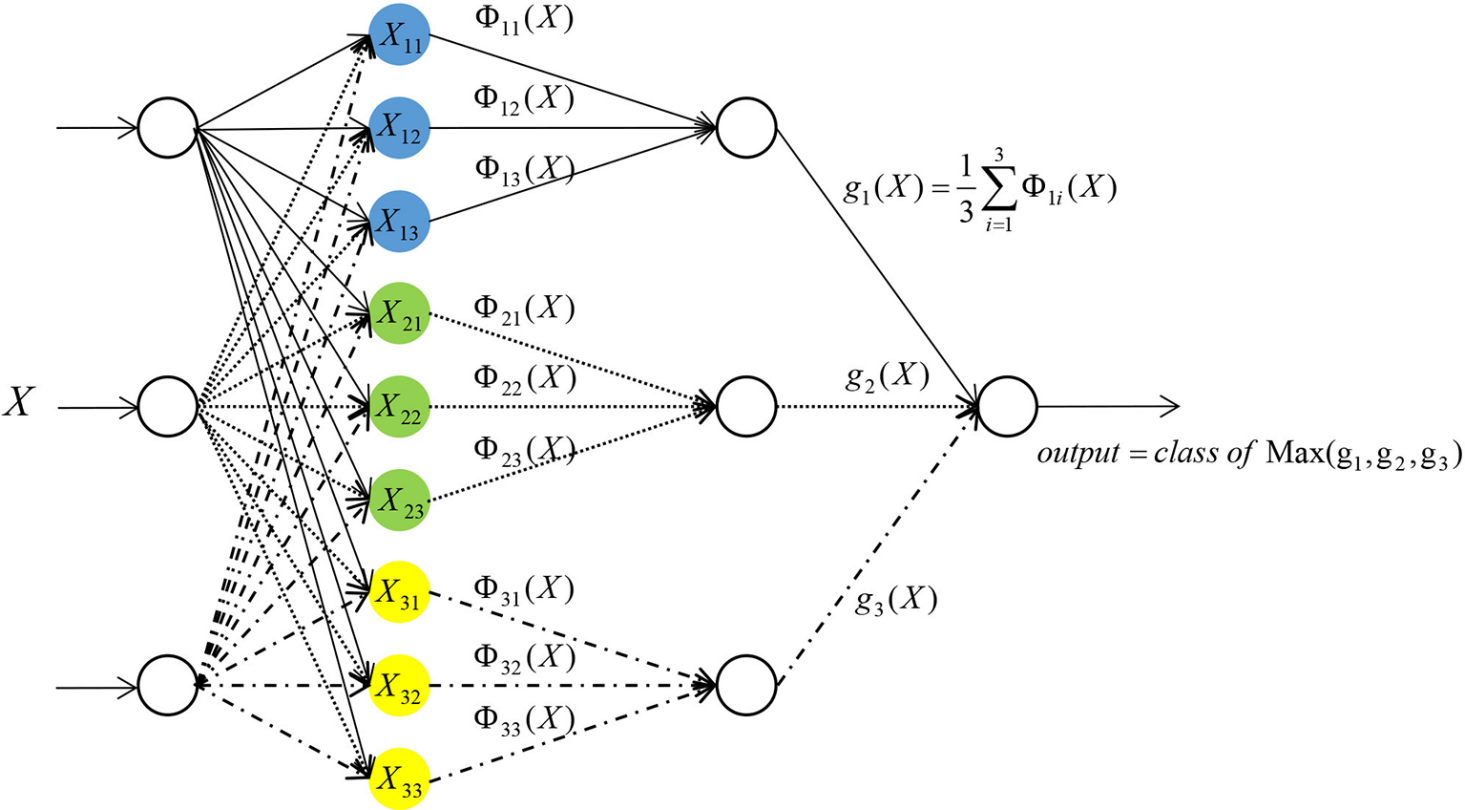
Network structure of probabilistic neural network.

PNN was used in this study (6–10). PNN is composed of four layers: input layer, radial base layer (pattern layer), decision-making layer (summation layer) and output layer (**Figure 1**).

Taking the network input vector dimension of three as an example, the details of each layer are outlined below.

The first layer is the input layer, which receives the input of samples X = (x_1_, x_2_, x_3_), where x_i_ (i = 1, 2, 3) represents the input of the number i neuron in this layer, and transmits the input data to the radial base layer. The number of neurons in this layer is equal to the number of feature variables.

The second layer is the pattern layer. The number of neurons in this layer is equal to the number of training samples. Each neuron in the pattern layer has a center. After receiving data from the input layer, the distance between the input data and each center is calculated. Each neuron will output a scalar. After the sample vector X is input into the pattern layer, the input-output relationship of the number j neuron in the class i mode of the pattern layer is determined by Formula (2):

(2) $$\Phi_{ij}=\exp\left(-\frac{\|X-X_{ij}{\|}^{2}}{\sigma^{2}}\right)$$

In the following formula, i = 1, 2, …, m, where m stands for the total number of training samples. In this study, m = 2. Xij stands for the center of the j^th^ neuron in the i^th^ class sample. *Φ_ii_* stands for the output of the j^th^ neuron in the i^th^ class sample. *σ* is a constant, which is the width parameter or smooth factor of the Gaussian curve. This constant plays an important role in the performance of PNN.

The third layer is a summation layer. The number of neurons in this layer is equal to the number of classification categories. In this study, the number of neurons in the summation level is two. Since each neuron in the pattern layer has been designated to a certain class, the neurons belonging to the same class in the pattern layer will be connected with the same neuron in the summation layer. While the neurons of a different class in the pattern layer will not be connected to the same neuron in the summation layer. The output of neurons belonging to the same class in the pattern layer is weighted and averaged in the summation layer (17) by Formula (3):

(3) $$s_{i}=\frac{\sum_{j=1}^{n}\Phi_{ij}}{n}$$

In the above formula, s_i_ stands for the output of the i^th^ class in the summation layer, and n stands for the number of neurons in the i^th^ class.

The fourth layer is the output layer. The number of neurons in this layer is equal to the number of neurons in the summation layer. Each neuron in the summation layer will be connected to the neurons in the output layer with corresponding weights. In this study, all the weights are taken as one. That is, all the neurons are connected with the same weight. The output layer is based on Bayesian classification decision theory, where there will be competition among neurons. By receiving the output of the summation layer neurons and judging the values, the neuron with the maximum posterior probability is found in the output layer. The output of this neuron is one, and all other neuron outputs is zero.

#### Optimization of the Probabilistic Neural Network by a Simulated Annealing Algorithm

To improve the performance of the network, it is allowed to take different σ values in the pattern layer by the activation functions of different class of neurons. Formula (2) can transform into Formula (4) as shown below:

(4) $$\Phi_{ij}=\exp\left(-\frac{\|X-X_{ij}{\|}^{2}}{\sigma_{i}2}\right)$$

The steps for optimizing the PNN with SA-PNN are as follows:

Step 1: Establish a fitness function. The goal of this study is to improve the accuracy of identification of metastases in lymph nodes. Therefore, the minimum number of classification errors is expected. The error rate of the selected fitness function for classification is:

$$f\left(\sigma \right)=\frac{N_{\text{error}}}{N_{\text{sum}}}\times 100\%$$

In this formula, N_error_ stands for the number of incorrectly identified samples in the training sample set, and N_sum_ stands for the total number of training samples.

Step 2: Initialize parameters. For the initial solution *σ*_0_, calculate the corresponding fitness function value f(σ_0_) and set the initial temperature t = t_0_.

Step 3: Set the number of iterations as Count = 0.

Step 4: Calculated the increment *Δf* = *f*(*σ*) - *f*(*σ*_0_), after the feasible solution *σ* is randomly generated from the neighborhood

Step 5: Accept *σ* as the new current solution and considered the starting point for the next time (σ0= σ), if *Δf* <0. Once *Δf* ≥ 0, determine whether e^-^ *^Δf^* ^/^ *^t^* > *rand*(0,1) (t is the current temperature) is valid. If so, accept the solution, otherwise discard the solution and take the original solution as the next starting point.

Step 6: Set the number of iterations for Count= Count+1. If Count does not reach the maximum number, return to Step 4, or continue with the next steps.

Step 7: Anneal. Decrease t gradually, where t→0, then proceed to Step 3.

In the steps above, the parameters settings are as follows: initial solution *σ*_0_ is randomly generated, initial temperature T_0 =_ 100, number of iterations Count=1000. The annealing strategy is the most commonly adopted exponential annealing *t_k_* = *α t_k_*_-1_, where k is a positive integer and *k* ≥ 1, 0 < α < 1.

**Figure 2:**
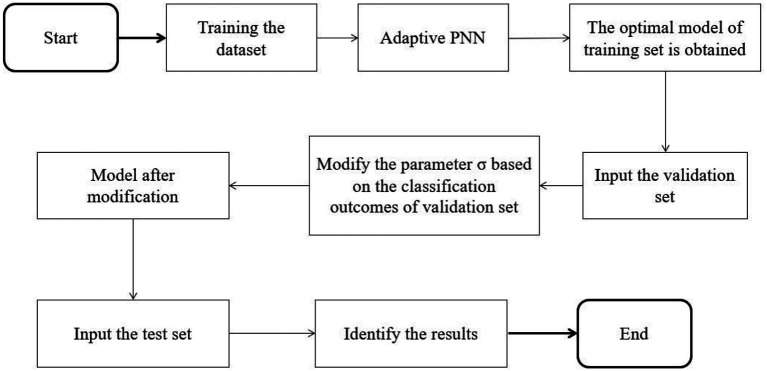
Flow chart of adaptive probabilistic neural network algorithm based on validation set.

Since the adaptive PNN is prone to over-fitting in the experimental process, a part of the data set is divided into a validation set to adjust the smooth factor to alleviate over-fitting. The specific algorithm flow is shown in **Figure 2**. When a group of σ values corresponding to the minimum value of the fitness function is discovered by a training set, validation is conducted with the validation set. The threshold is then set. When the classification accuracy of the validation set is lower than the threshold value, the obtained group of σ values is adjusted. The test set is then examined, which helps prevent over-fitting.

### Other Prediction Models

In this study, besides the SA-PNN, five algorithms, including the BP neural network, RBF neural network, the classify function, SVM and kNN, were applied to analyze the data. The data analysis for the BP neural network, the RBF neural network, the classify function, SVM and kNN was performed using MATLAB 2017 (MathWorks Inc., Natick, MA, USA).

### Calculation of the Classification Performance Evaluation Index

In this study, the accuracy, specificity and sensitivity of the classifier are considered in judging its performance (18). The formulas for sensitivity, specificity and accuracy are as follows:

$$SEN=\frac{TP}{TP+FN}\times 100\%$$

$$SPE=\frac{TN}{FP+TN}\times 100\%$$

$$ACC=\frac{TP+TN}{TP+FN+FP+TN}\times 100\%$$

SEN, SPE and ACC represent sensitivity, specificity, and accuracy, respectively. TP (true positivity) represents the number of tumor samples correctly identified, FN (false negativity) represents the number of tumor samples mistakenly identified as normal tissue samples, TN (true negativity) represents the number of normal tissue samples correctly identified, and FP (false positivity) represents the number of normal tissue samples mistakenly identified as tumor samples.

Through stratified random sampling, 60% of samples were selected for the training set from the data set of metastatic thoracic lymph nodes and non-metastatic thoracic lymph nodes in lung cancer patients, 20% of samples were selected for the validation set, and 20% of samples were selected for the test set. The experiment was repeated 20 times. The 20-hold-out method was used, and the average value of 20 results was taken as the final result.

## Results

### Sample Information and Dielectric Properties of Lymph Nodes

By combining current and previously published data (19), the dielectric parameters from 41 lung cancer metastatic thoracic lymph nodes and 178 non-metastatic lung thoracic lymph nodes from 74 patients were measured (**Table 1**) using an open-ended coaxial probe with 3,951 frequency points in the range of 50 MHz to 4 GHz.

**Table 1:** The relevant information of samples.

Items	Value
Number of patients	74
Age of patients	30 ~ 82
number of metastatic thoracic lymph nodes	178
number of non-metastatic thoracic lymph nodes	41
Temperature of samples (°C);	23.5 ± 1.7

**Figure 3:**
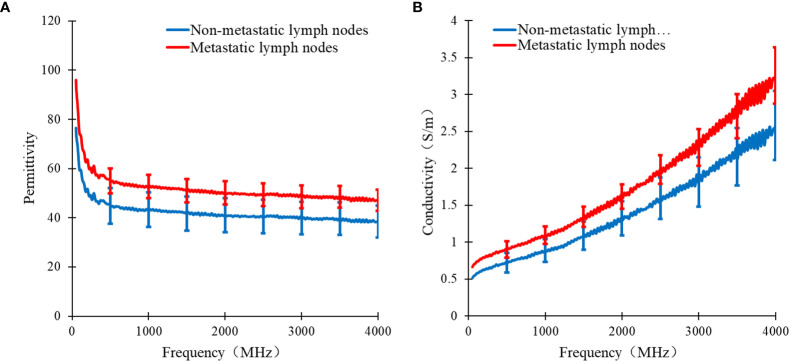
Curve of median dielectric properties of lung tissue and lymphonodi pulmonales in the range of 50MHz~4GHz. Curve of **(A)** the median relative permittivity **(B)** the median conductivity with metastatic and non-metastatic lymphonodi pulmonales of lung cancer.

In **Figure 3**, the curve of the median of the dielectric properties of metastatic and non-metastatic thoracic lymph nodes in lung cancer patients is shown. From the measurements, it can be learned that there are obvious differences in the permittivity and conductivity between metastatic and non-metastatic thoracic lymph nodes in the range of 50 MHz to 4 GHz. The permittivity and conductivity of metastatic thoracic lymph nodes are higher than those of non-metastatic thoracic lymph nodes.

### SD-1 Values of Permittivity and Conductivity of Lymph Nodes

**Figure 4:**
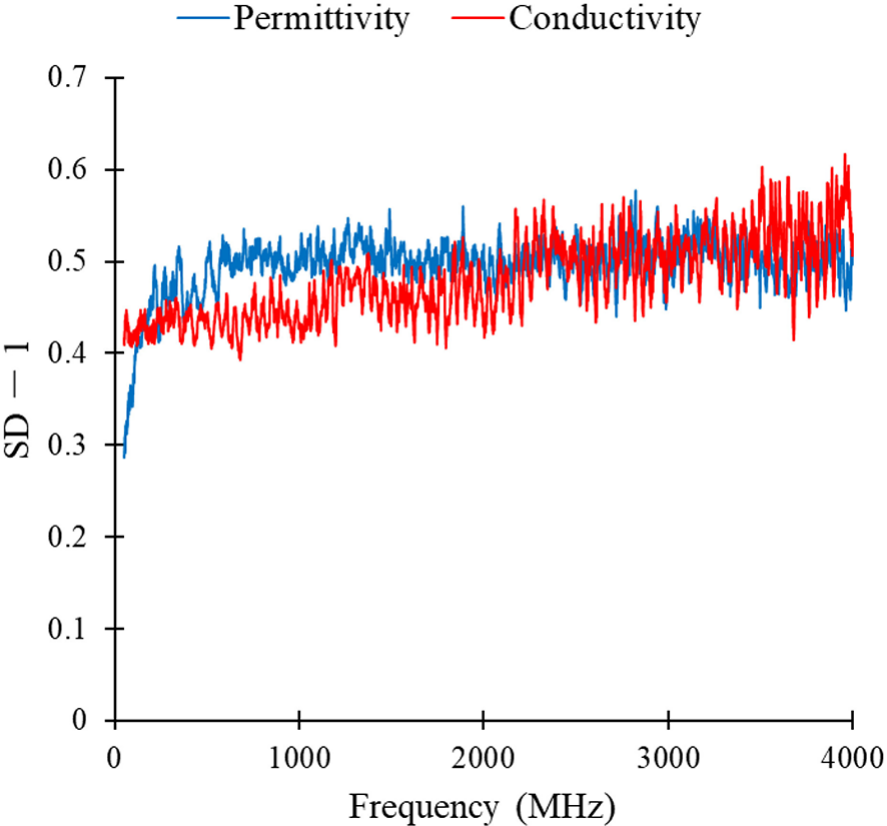
SD-1 values of permittivity and conductivity of lung lymphonodi pulmonales at various frequency points.

The SD-1 values of permittivity and conductivity for pulmonary thoracic lymph nodes at each frequency point are shown in **Figure 4**. Among the top 100 values with the highest feature scores, only the 65^th^ has a permittivity at 2824 MHz, with the rest being conductivity scores. Therefore, it can be preliminarily inferred that using conductivity as a feature parameter to differentiate metastatic and non-metastatic thoracic lymph nodes in lung cancer patients is more effective.

### Classification Results for SA-PNN at Different Frequencies

**Figure 5:**
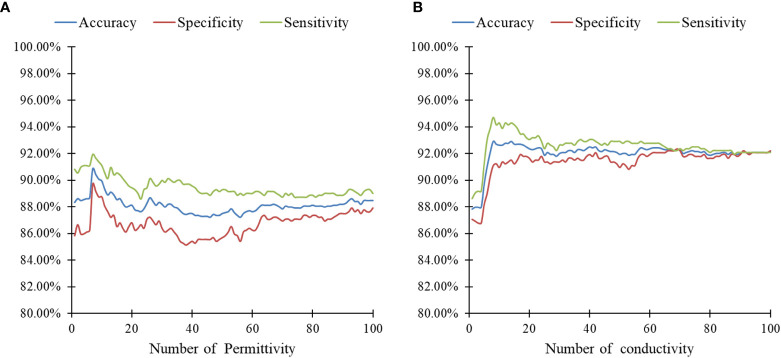
Results of SA-PNN classification of lymphonodi pulmonales. **(A)** permittivity and **(B)** conductivity at different number frequencies.

The classification results of SA-PNN using dielectric parameters at different frequency points as feature vectors is shown (**Figure 5**). The highest differentiating accuracy rate of 90.83% was achieved when the permittivity at seven frequencies (2824, 2799, 2798, 2823, 2821, 2819, and 1888 MHz) were invoked as feature parameters (**Figure 5A**). The corresponding specificity and sensitivity values were 91.94% and 89.72%, respectively. The highest differentiating accuracy rate of 92.92% was achieved when the conductivity at eight frequencies (3959, 3958, 3960, 3978, 3510, 3889, 3888, and 3976 MHz) were invoked as feature parameters (**Figure 5B**). The corresponding specificity and sensitivity values were 94.72% and 91.11%, respectively.

These results also support the previous speculation of the SD-1 value using conductivity as a feature with better differentiation between metastatic and non-metastatic thoracic lymph nodes. Therefore, for the identification of metastatic and non-metastatic thoracic lymph nodes in lung cancer patients, the conductivity at eight frequency points (3959, 3958, 3960, 3978, 3510, 3889, 3888, and 3976 MHz) was finally selected as the feature vector.

### Comparison of the Identification Results Among Six Algorithms

**Figure 6:**
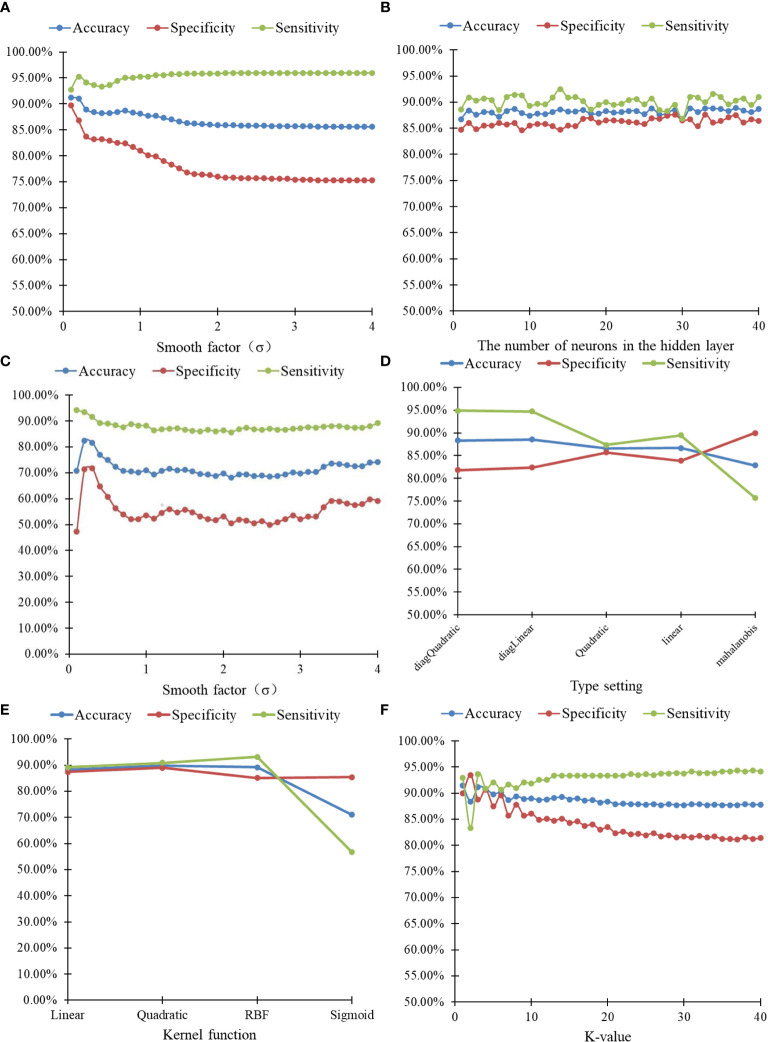
20-hold-out validation results of lymphonodi pulmonales by six algorithms with different parameters. **(A)** PNN. **(B)** BP neural network. **(C)** RBF neural network. **(D)** Classify. **(E)** SVM. **(F)** kNN.

The identification results of thoracic lymph nodes by PNN, BP neural network, RBF neural network, the classify function, SVM and kNN algorithms under different parameters are shown in **Figure 6**. For PNN, when the smooth factor σ=0.1, the highest differentiating accuracy rate of 91.25% was achieved, and the specificity and sensitivity were 92.78% and 89.72%, respectively. For the BP neural network, when the number of neurons in the hidden layer was 37, the highest differentiating accuracy rate of 88.89% was achieved, and the specificity and sensitivity were 90.28% and 87.50%, respectively. For the RBF neural network, when the smooth factor σ = 0.2, the highest differentiating accuracy rate of 82.43% was achieved, and the specificity and sensitivity were 93.47% and 71.39%, respectively. For the classify function, when the type setting was “diagLinear,” the highest differentiating accuracy rate of 88.54% was achieved, and the specificity and sensitivity were 94.72% and 82.36%, respectively. For SVM, when the kernel function was “Quadratic,” the highest differentiating accuracy rate of 89.93% was achieved, and the specificity and sensitivity were 90.83% and 89.03%, respectively. For the kNN algorithm, when the k-value =1, the highest differentiating accuracy rate of 91.46% was achieved, and the specificity and sensitivity were 92.92% and 90.00%, respectively.

## Discussion

In this study, the dielectric parameters of 219 lymph nodes (178 non-metastatic and 41 metastatic) from 74 patients were measured. The number ratio of metastatic and non-metastatic lung lymph node data was about 1:4, with a significant class imbalance. In machine learning, class imbalance often affects the performance of the trained classifier, which causes certain class bias in identification for the classifier. Therefore, to obtain an objective classifier, this study used the SMOTE algorithm to pre-process the lymph node data. The basic idea of SMOTE algorithm is to generate synthetic examples, by taking each minority class sample as center, calculating its k nearest neighbors. Randomly select a sample from its k nearest neighbors, connect this sample with the center one, and then randomly select a point along the line segment between two points as a new minority class sample (15). The conductivity values at eight frequency points (3959, 3958, 3960, 3978, 3510, 3889, 3888, and 3976 MHz) were selected as the feature vector. The classification of metastatic and non-metastatic thoracic lymph nodes in lung cancer patients based on dielectric properties is studied by the proposed adaptive probabilistic neural network, and the best classification results of several methods are summarized in **Table 2**. As shown in **Table 2**, the SA-PNN proposed in this paper achieved the highest classification accuracy, 92.92%, which indicates that its differentiation performance is higher than other classification algorithms.

**Table 2:** The best classification results of lymphonodi pulmonales by different methods.

Methods	Accuracy (%)	Sensitivity (%)	Specificity (%)
PNN	91.25	92.78	89.72
BP	88.89	90.28	87.50
RBF	82.43	93.47	71.39
Classify	88.54	94.72	82.36
SVM	89.93	90.83	89.03
kNN	91.46	92.92	90.00
SA-PNN	92.92	94.72	91.11

With the popularity of lung cancer screening, the number and proportion of people diagnosed with early-stage disease is increasing. Surgery is considered the most effective treatment of early-stage lung cancer (20). With the various surgical management techniques of early-stage lung cancer, lymph node staging is considered an important criterion for these resections (21). At present, the diagnosing thoracic lymph nodes are required to go through multiple processing steps, such as tissue sectioning and staining, which takes a long time and is time sensitive. Therefore, a simple operational, rapid determination and noninvasive auxiliary diagnostic method for the identification of malignant tumors in surgery is needed.

At present, there are few published studies focusing on the method of tissue classification based on dielectric properties. Among the few known papers, most of them model the fit for dielectric parameter data of tissue samples in a wide frequency band. The fitted model parameters were taken as feature vectors, follow by classification by SVM (22–26), linear discriminant analysis (27), kNN (28), BP neural network (29), and RBF neural network (29). When processing data, the model parameters obtained by data fitting have certain volatility, which affects the identification results. In addition, the time cost will be increased when the dielectric parameters of samples are measured in a wide frequency band and complicated data fitting is required. To optimize data measurement and processing times, this paper analyzed and obtained the dielectric parameters of representative frequency points as the feature vector. This is clearly more convenient for future applications. Within the classification methods, the kernel function and its parameters of SVM, the k-value of the kNN algorithm, the number of hidden layers and neurons in the BP neural network, and the smooth factor of the radial basis function in the RBF neural network play key roles in the performance of each algorithm. However, it was quite challenging to select these parameters properly. Compared with the aforementioned algorithms, the number of neurons in each layer of the adaptive PNN proposed in this paper is easy to determine. The smooth factor of network parameters can modify adaptively, which can maximize its classification performance. This is also the advantage of adaptive PNN compared with other algorithms.

The study of differentiation between benign and malignant tissues during surgery is an important clinical application of biological tissue dielectric measurements, which can provide auxiliary diagnostic methods for the identification of malignant tumors during surgery. The main purpose of this paper is to improve the pattern recognition module of real-time detection and identification systems of benign and malignant tissues based on dielectric properties of tissues. At present, the collected number of sample data is relatively limited. More data is required for the training dataset of the model in practical clinical applications, in order to obtain a model with higher classification accuracy. It is necessary to collect more data to achieve a more reliable model. In addition, the current data of tissue dielectric properties are measured *in vitro*. The dielectric properties of tissue *in vitro* cannot completely represent the dielectric properties of *in vivo* tissue because the moisture content and temperature of tissue *in vitro* will be different from that of *in vivo* tissue. These differences would also affect the measured results. Therefore, follow-up studies should include a large number of real-time *in vivo* tissue dielectric property data for classification research.

## Data Availability Statement

The raw data supporting the conclusions of this article will be made available by the authors, without undue reservation.

## Ethics Statement

The studies involving human participants were reviewed and approved by the ethics committee of Nanfang Hospital, Southern Medical University, Guangzhou, China (NFEC-2017-070). The patients/participants provided their written informed consent to participate in this study.

## Author Contributions

DL and KC designed the study. DL, HY, ZW, and ZC were primarily responsible for conceptualization, methodology, and writing – reviewing and editing. HY, ZW, and ZC were responsible for data curation, software, and writing – original draft preparation. JF, XL, JZ, and HW were responsible for data revision. XY and KC revised the manuscript. DL, HY, ZW, and ZC contributed to this study equally. All authors contributed to the article and approved the submitted version.

## Funding

This project was supported by the Science and Technology Program of Guangzhou, China (201704020091) and the Dean Research Funding of Nanfang Hospital, Southern Medical University, China (2016B018) and Nanfang Thoracic Surgery collaborative project (NFTS-T-0202).

## Conflict of Interest

The authors declare that the research was conducted in the absence of any commercial or financial relationships that could be construed as a potential conflict of interest.

## References

[1] Asami K. Dielectric properties of microvillous cells simulated by the three-dimensional finite-element method. Bioelectrochemistry (Amsterdam Netherlands) (2011) 81(1):28–33. 10.1016/j.bioelechem.2011.01.002 21333613

[2] Wang H He Y Yang M Yan Q You F Fu F. Dielectric properties of human liver from 10 Hz to 100 MHz: normal liver, hepatocellular carcinoma, hepatic fibrosis and liver hemangioma. Biomed Mater Eng (2014) 24(6):2725–32. 10.3233/bme-141090 25226977

[3] Cheng Y Fu M. Dielectric Properties for Differentiating Normal and Malignant Thyroid Tissues. Med Sci Monit Int Med J Exp Clin Res (2018) 24:1276–81. 10.12659/msm.908204 PMC5844190 29499032

[4] Summers PE Vingiani A Di Pietro S Martellosio A Espin-Lopez PF Di Meo S. Towards mm-wave spectroscopy for dielectric characterization of breast surgical margins. Breast (Edinburgh Scotland) (2019) 45:64–9. 10.1016/j.breast.2019.02.008 30884340

[5] Zhou DF Zhai WK Sun Y Han S Huang LM Xin XG. [Differences in dielectric properties between mucosal and serosal surface of malignant colorectal tissues, adjacent tissues at 1 cm and 3 cm and normal colorectal tissues]. Nan Fang Yi Ke Da Xue Xue Bao (2018) 38(4):434–42. 10.3969/j.issn.1673-4254.2018.04.11 PMC6765664 29735444

[6] Mohamed Shakeel P Desa MI Burhanuddin MA. Improved watershed histogram thresholding with probabilistic neural networks for lung cancer diagnosis for CBMIR systems. Multimed Tools Appl (2020) 79(23):17115–33. 10.1007/s11042-019-7662-9

[7] Daqrouq K Chen S Khalaf E Morfeqa A Sheikha M Qatawneh A. Wavelet entropy based probabilistic neural network for classification. Curr J Appl Sci Technol (2019) 35(5):1–7. 10.9734/cjast/2019/v34i530145

[8] Woźniak M Połap D Capizzi G Sciuto GL Kośmider L Frankiewicz K. Small lung nodules detection based on local variance analysis and probabilistic neural network. Comput Methods Programs Biomed (2018) 161:173–80. 10.1016/j.cmpb.2018.04.025 29852959

[9] Ahmadlou M Adeli H. Enhanced probabilistic neural network with local decision circles: A robust classifier. Integr Comput Aided Eng (2010) 17:197–210. 10.3233/ICA-2010-0345

[10] Gaikwad SBJM. Brain tumor classification using principal component analysis and probabilistic neural network. Int J Comput Appl (2015) 120(3):5–9. 10.5120/21205-3885

[11] Samuel RK Venkumar P. Optimized Temperature Reduction Schedule for Simulated Annealing Algorithm. Mater Today: Proc (2015) 2(4):2576–80. 10.1016/j.matpr.2015.07.209

[12] Mafarja MM Mirjalili S. Hybrid Whale Optimization Algorithm with simulated annealing for feature selection. Neurocomputing (2017) 260:302–12. 10.1016/j.neucom.2017.04.053

[13] Bobowski JS. Permittivity Measurements of Biological Samples by an Open-Ended Coaxial Line. Prog In Electromagn Res B (2012) 40:159–83. 10.2528/pierb12022906

[14] Liu Y Huang Y Xin X Yu X. [Sensing volume of tissue dielectric property measurement with open-ended coaxial probe]. Nan Fang Yi Ke Da Xue Xue Bao (2020) 40(7):1036–43. 10.12122/j.issn.1673-4254.2020.07.19 PMC7386231 32895168

[15] Chawla NV Bowyer KW Hall LO Kegelmeyer WP. SMOTE: Synthetic Minority Over-sampling Technique. J Artif Intell Res (2002) 16(1):321–57. 10.1613/jair.953

[16] Pohjalainen J Räsänen O Kadioglu S. Feature selection methods and their combinations in high-dimensional classification of speaker likability, intelligibility and personality traits. Comput Speech Lang (2015) 29(1):145–71. 10.1016/j.csl.2013.11.004

[17] Lin D-T. Target Components Discrimination using Adaptive Time-Delay Neural Network. J Inf Sci Eng (2004) 20(5):959–80s. 10.1186/s12868-016-0283-6

[18] Yang M Gu XN Liu F Wang YY Cai YL Liu DM. [Diagnostic values of breast imaging and reporting data system and ultrasonic elastography for benign and malignant breast lesions]. Zhonghua Yi Xue Za Zhi (2013) 93(39):3125–7. mdl-24417992 24417992

[19] Yu X Sun Y Cai K Yu H Zhou D Lu D. Dielectric Properties of Normal and Metastatic Lymph Nodes Ex Vivo From Lung Cancer Surgeries. Bioelectromagnetics (2020) 41(2):148–55. 10.1002/bem.22246 31912926

[20] Tandberg DJ Tong BC Ackerson BG Kelsey CR. Surgery versus stereotactic body radiation therapy for stage I non-small cell lung cancer: A comprehensive review. Cancer (2018) 124(4):667–78. 10.1002/cncr.31196 29266226

[21] Ginsberg RJ Rubinstein LV. Randomized trial of lobectomy versus limited resection for T1 N0 non-small cell lung cancer. Lung Cancer Study Group. Ann Thorac Surg (1995) 60(3):615–22; discussion 22-3. 10.1016/0003-4975(95)00537-u 7677489

[22] Truong BCQ Tuan HD Wallace VP Fitzgerald AJ Nguyen HT. The Potential of the Double Debye Parameters to Discriminate Between Basal Cell Carcinoma and Normal Skin. IEEE Trans Terahertz Sci Technol (2015) 5(6):990–8. 10.1109/TTHZ.2015.2485208

[23] Grewal PK GF. Pilot study: electrical impedance based tissue classification using support vector machine classifier. IET Sci Meas Technol (2014) 8(6):579–87. 10.1049/iet-smt.2013.0087

[24] Yilmaz T Kılıç MA Erdoğan M Çayören M Tunaoğlu D Kurtoğlu İ. Machine learning aided diagnosis of hepatic malignancies through in vivo dielectric measurements with microwaves. Phys Med Biol (2016) 61(13):5089–102. 10.1088/0031-9155/61/13/5089 27321132

[25] Truong BC Tuan HD Fitzgerald AJ Wallace VP Nguyen HT. A dielectric model of human breast tissue in terahertz regime. IEEE Trans Biomed Eng (2015) 62(2):699–707. 10.1109/tbme.2014.2364025 25347869

[26] Song H Sato H Koide T Arihiro K Okada M Kadoya T. “Breast tumor tissues classification using the modified cole-cole parameters with machine learning technique”. In: 12th European Conference on Antennas and Propagation (EuCAP 2018) IET (2018). p. 1–2.

[27] da Silva JE de Sá JP Jossinet J. Classification of breast tissue by electrical impedance spectroscopy. Med Biol Eng Comput (2000) 38(1):26–30. 10.1007/bf02344684 10829386

[28] Saçlı B Aydınalp C Cansız G Joof S Yilmaz T Çayören M. Microwave dielectric property based classification of renal calculi: Application of a kNN algorithm. Comput Biol Med (2019) 112:103366. 10.1016/j.compbiomed.2019.103366 31386972

[29] Helwan A Idoko JB Abiyev RH. Machine learning techniques for classification of breast tissue. Proc Comput Sci (2017) 120:402–10. 10.1016/j.procs.2017.11.256

